# Supplementary material for: Are carbon emissions trading and green financial instruments synergistic? -Comprehensive quantitative research based on content analysis
Source: PLoS One. 2024 Mar 7;19(3):e0298601. doi: 10.1371/journal.pone.0298601 (PMC10919601; doi:10.1371/journal.pone.0298601)
Supplement: S1 Data — (ZIP) [file pone.0298601.s001.zip › Data/地级市Z_F工作报告.zip!/╜¡╦╒/╤∩╓▌╩╨/└┤╫╘╣1⁄2╓┌║┼í╛┬φ┐╦╩2╛▌═°í┐_2008.docx]

各位代表：

现在，我代表市人民政府向大会报告工作，请予审议，并请市政协各位委员和其他列席人员提出意见。

过去五年和2007年政府工作的回顾

本届政府任期的五年，是我市经济加快发展，综合实力显著增强的五年；是城市功能不断完善，城乡面貌发生重大变化的五年；是各项事业协调发展，和谐社会扎实推进的五年；也是人民群众得到更多实惠，全面小康建设成效明显的五年。五年来，我们在中共扬州市委的领导下，坚持邓小平理论和“三个代表”重要思想，贯彻落实科学发展观，依靠和带领全市人民，拼搏奋进，开拓创新，圆满完成了市五届人大历次会议确定的任务。

一、综合经济实力进一步壮大

2007年全市预计实现地区生产总值1311亿元，按可比价比上年增长15.7%，是2002年的2.4倍；人均地区生产总值29000元。实现财政总收入213.6亿元，其中一般预算收入85.7亿元，分别增长35.2%和36%，是2002年的3.8倍和3.7倍。完成全社会固定资产投资715亿元，增长34.1%，是2002年的4倍。

工业经济快速发展。大力实施工业第一方略，全力推进“双创”和“三重”，规模以上工业产值五年年均增长30.8%。预计2007年全市规模以上工业产值2580亿元、增加值710亿元、利税210亿元，分别增长35%、21.3%和38%，是2002年的3.8倍、3.9倍和4.2倍。石油化工、汽车及零部件、船舶、装备制造等支柱产业占规模以上工业总量60%以上。国内自主品牌荣威轿车在上汽仪征基地实现批量生产。太阳能光伏、半导体照明等新兴产业蓬勃发展。产值超10亿元企业30家，其中50亿元以上4家，分别比2002年增加24家和2家。完成工业投资502亿元，增长39.2%。实施亿元以上项目200个，其中10亿元以上项目10个，比2002年增加184个和9个。拥有省级品牌223个、国家级品牌29个，比2002年增加173个和27个。建筑业长足发展。2007年总产值703亿元，五年年均增长25%，累计获得鲁班奖20项。

农村经济持续增长。扎实推进新农村建设，大力发展现代高效农业。预计2007年农业增加值101亿元，是2002年的1.5倍。4大农业特色板块和115个高效农业园区加快建设，海峡两岸（扬州）农业合作试验区建设全面启动。2007年全市新增高效农业面积41.7万亩，累计149万亩。五年改造中低产田62万亩。粮食保持稳定增产。2007年总产266万吨，比2002年增长26%。优质粮油、蔬菜、畜禽、水产、林木等五大加工企业集群初步形成。市级以上农业龙头企业60家，实现销售130亿元，分别是2002年的2倍和3.5倍。农产品质量稳步提高。累计发展“三品”品牌554个，高邮鸭蛋、宝应荷藕列为国家原产地保护产品。全面完成“菜篮子”工程建设任务。建成新农村示范村10个、全面小康村432个。水利、粮食流通、农业开发、农机、气象等工作得到加强。

服务业发展水平稳步提高。加快发展现代服务业，巩固提升传统服务业。预计2007年服务业增加值462亿元，社会消费品零售总额416亿元，分别增长16.1%和17%，是2002年的2.5倍和2.1倍。港口、公铁水、石化、商贸等重点物流园区建设加快，已有一批企业入驻经营。全市港口货物吞吐量5516万吨，集装箱吞吐量达28万标箱。加大信息基础设施投入力度，信息服务业基地加快建设。全年信息服务业收入46亿元，增长17.9%。实施旅游名城建设行动计划，全面提升旅游业质态。个园、何园、双博馆升为4A级景点，新建了万花园等一批景点，开通了古运河水上游览线。全市接待境内外游客1556万人次，实现旅游总收入155.8亿元，是2002年的2.4倍和3倍。“三把刀”行业焕发活力，“扬州师傅”品牌进一步做响，6家企业获“中华老字号”称号。注重培育新型业态，一批商贸重点项目竣工开业。全市金融机构年末存贷款余额分别为1276.3亿元和765.9亿元，增量贷存比达95%。招商银行扬州分行挂牌营业。保险业健康发展，保险机构由2002年的8家增加到31家。五年累计完成房地产投资345亿元，商品房竣工面积1300万平方米。

科技创新成效明显。加大政策引导扶持力度，推进创新体系建设。2007年组织实施省级以上科技项目440个，比2002年增加121个。建成市级以上农业科技示范园47个。五年引进推广农业新品种200多个。已建成省级以上高新技术企业317家，省级以上研发机构40家，分别比2002年增加250家和18家。实现高新技术产业产值580亿元，增长36%，是2002年的7.8倍。汽车及零部件、金属板材加工及设备制造和半导体照明被列为国家级产业基地。深入推进与中科院、南京大学等一批科研单位和高校的合作，中科院应用技术研发与产业化中心等机构来扬落户。

科技创新公共服务平台、企业孵化器建设以及人才引进培养工作稳步推进。专利授权1438件，是2002年的3.7倍。

二、改革开放实现新突破

各项改革不断深化。五年来，基本完成了工业、商贸流通、建筑企业的产权制度改革。实施市直工业、商贸流通管理体制和生产经营型事业单位改革。成立市国有资产监督管理委员会和金融办公室。组建了城建、交通、商贸和旅游等投融资平台。宝胜股份、江苏琼花、联环药业先后上市，5家上市公司完成股权分置改革。成立一批中小企业担保机构和2家创投公司。农村综合改革稳步推进。各项支农惠农政策得到落实。全面免征农业税及其附加。启动政策性农业保险。农村“三大合作”组织发展加快。新一轮镇村区划调整取得实效。基本农田保护工作得到加强，土地有偿使用制度改革不断深化。土地复垦力度加大，实现了耕地占补动态平衡。

开发开放步伐加快。坚持集聚集约发展，沿江开发取得突破，沿河开发加快推进，园区建设成效明显。新增3家省级经济开发区，国家级出口加工区建成运行。“八区二园”累计完成基础设施投入162亿元，已成为经济发展的重要增长极。坚持举全市之力招商引资。精心举办“烟花三月”国际经贸旅游节，积极开展赴欧美、日韩、港台等境内外招商活动。2007年全市协议利用外资33亿美元，注册外资实际到账11.45亿美元，分别增长35%和50%。五年实际利用外资36.8亿美元，是前五年的6倍。近30家世界500强企业来扬落户，一批投资过亿美元项目投入运营。外贸外经快速发展。2007年出口总额31亿美元，外经营业额1.5亿美元，分别是2002年的3.8倍和2.4倍。外事、侨务、台湾事务工作取得新成绩。

民营经济加快发展。深入实施鼓励全民创业的各项政策措施，民营资本投资领域更加广泛，投资规模不断扩大。2007年新发展民营企业9998家，新增民资注册资本金316亿元，分别增长24%和47%；五年累计达3.8万家和850亿元，是前五年的2.4倍和2.2倍。民营经济对全市地区生产总值和税收的贡献份额分别达49%和60%，比2002年提高5个和30个百分点。

三、城乡面貌发生重大变化

基础设施建设取得突破性进展。宁启铁路扬州段、润扬长江公路大桥、西北绕城高速公路、沿江高等级公路、安大公路三垛以南段等交通骨干工程建成通车。海通都公路江都段、扬天公路仪征段实现贯通，金宝南线等干线公路正在加紧施工。到2007年底，全市高速公路通车里程达241公里，公路通车总里程9247公里，其中新改建农村公路4800公里，行政村等级公路通达率100%。扬州港“一港三区”加快建设，3号、4号泊位投入营运，5号泊位开工建设。南水北调东线一期、京杭运河“三改二”等工程进展顺利。完成扬州电厂扩建、二电厂二期工程。五年建设110千伏以上变电站25座。市区10万用户实施天然气置换。扩建了扬州四水厂，21个近郊乡镇实现区域集中供水。

城乡环境明显改善。推进城乡规划全覆盖。修编了城市总体规划，完成“一体两翼”概念性规划和城镇体系规划，编制了古城保护、新城西区、沿江地区等控制性详规，以及城市绿地系统和“大江风光带”等专项规划。新城西区建设成效显著，蜀冈-瘦西湖风景区建设力度加大，广陵新城启动建设，市开发区生活配套功能逐步完善。市区建成区面积从2002年的63平方公里增加到98平方公里。五年新改建城市道路128条、桥梁36座，整治河道11条，改造街巷258条。加大古城保护力度。实施了吴道台宅第、卢氏古宅、汪鲁门故居等整修工程，盐宗庙、大王庙建成开放，“双东”历史街区等项目稳步推进，整治了古城沿街建筑风貌，名城特色进一步彰显。各县城镇、重点中心镇建设步伐加快，城镇设施配套水平和综合服务功能明显增强。农村环境综合整治取得新成效。2007年全市城市化率达51.5%，比2002年提高8.8个百分点。首创全国中小城市数字化管理新模式，城管责任体系进一步完善，环卫设施建设得到加强，城市管理水平不断提高。

生态市建设扎实推进。编制市、县生态建设规划，出台《生态市建设行动计划》。全面推进森林生态体系建设。实施“绿杨城郭新扬州”工程，新建漕河、邗沟风光带和润扬、蜀冈等森林公园，提升古运河风光带建设水平。五年市区新增绿化面积776万平方米。全市森林覆盖率从2002年的8.2%提高到14.5%。各县（市）及邗江区创成国家生态示范区。加大水和空气污染防治力度。市区及各县（市）污水处理厂相继建成运营，工业废水排放达标率提高到82%。空气质量稳定在二级以上。关停了70家小化工企业。2007年全市万元地区生产总值综合能耗下降4%，化学需氧量、二氧化硫排放量分别下降3.8%和3.2%，实现了节能减排的预定目标。经过全市上下共同努力，我市先后荣获“国家园林城市”、“中国人居环境奖”和“联合国人居奖”。

四、社会事业与精神文明建设全面加强

教育事业加快发展。城乡教育布局不断优化。义务教育普及水平持续巩固，高中阶段毛入学率由2002年的62.5%提高到95.7%。全部免除义务教育阶段学杂费，落实了对困难学生的“一免一补”政策。以“宏志班”为代表的集群式帮扶形成特色。素质教育稳步实施，教育信息化步伐加快，教育质量不断提高。高考本二以上万人口上线率达22.2。职业教育进一步发展，中等职业学校招生占高中阶段招生总数的52%。建成扬子津科教园区、扬州职大新校区和10所国家级重点职业学校，建立国家级技能型紧缺人才培养培训基地4个、省级基地9个。

公共卫生服务体系进一步健全。建成城乡社区卫生服务中心67个、社区卫生服务站553个。办好惠民医院和惠民门诊，落实单病种限价等措施，群众看病难、看病贵问题得到缓解。加强疾病预防工作，传染病得到有效控制。医疗卫生设施建设取得积极进展，苏北医院、市妇幼保健院和市急救中心等建设工程加快实施。建成市医疗废物处理中心，市区和近郊实施了集中无害化处理。全年农村新增无害化户厕4.6万个。建成国家级卫生镇3个、省级卫生镇29个。

文化扬州建设深入推进。制订《扬州文化博览城建设规划纲要》。实施“名城解读”工程，市双博馆和各县（市）博物馆以及中国扬州剪纸博物馆、崔致远纪念馆等建成开放。瘦西湖及扬州历史城区列入申报世界文化遗产预备名单，扬州被确定为中国大运河联合申遗牵头城市。扬剧、清曲、漆器和玉雕等7个项目被列为首批国家级非物质文化遗产。新增全国重点文物保护单位6处。新改建乡镇文化站42家，建成农家书屋379个。农民艺术节、社区艺术节和“市民日”等群众性文化活动日益丰富。文化艺术创作取得新成绩。电视剧《江塘集中营》获全国“五个一工程”奖，扬剧《真假24小时》、木偶剧《白雪公主》获省优秀剧目一等奖。成功举办首届世界运河名城博览会暨运河名城市长论坛。承办了第十八届亚乒赛和全国十运会艺术体操、男子举重等一批重大赛事。

精神文明建设不断加强。制定并实施《城市文明建设行动计划》。深入开展社会主义荣辱观、文明礼仪教育和未成年人思想道德建设工作，广泛开展“三下乡”和“四进社区”活动，推进了文明社区、文明村镇、文明行业的创建。电影《江北好人》弘扬了扬州人的良好道德风尚。广泛宣传刘应启、吴杰、陈巧云、葛新峰等一批先进典型，促进了社会文明程度的进一步提高。计划生育工作得到加强，2007年人口出生率为5.7％。。关心下一代、老龄和残疾人事业、仲裁、档案方志、新闻出版等工作取得新进展。

五、和谐社会建设取得明显成效

人民生活不断改善。2007年城市居民人均可支配收入和农民人均纯收入预计分别达15000元和6500元，比上年增长16%和11.8%，是2002年的1.9倍和1.7倍。人均教育文化娱乐支出年均增长17.6%。逐步健全多层次的住房供应保障体系。农民人均住房使用面积40.9平方米，城市居民人均居住面积35.5平方米，均超过省全面小康标准。2007年市区开工建设限价商品房和解危解困房20.9万平方米，发放低收入家庭购房补贴324户，廉租房保障户1166户。五年全市完成农村草危房改造3714户。

社会保障体系逐步健全。坚持每年下发“一号文件”，部署改善民生工作。2007年全市采集就业岗位13.5万个，职业技能培训8万人次，新增就业4.5万人。城镇登记失业率3.1%，充分就业社区达标率为68%。养老、医疗、失业、工伤、生育五大保险扩面续保不断扩大，参保人数分别达59万、75万、51万、45万和35万人。被征地农民参加基本生活保障人数达4万人。建立城镇居民医疗统筹保险。新型农村合作医疗覆盖率达95%。城乡低保实现应保尽保。对低保边缘群体和困难家庭实施临时救助等制度。农村五保户集中供养率达70%。城乡困难群众医疗救助扎实开展。慈善事业发展迅速。

社会保持和谐稳定。完善维稳、调处和防控机制，加强社会治安综合治理，平安扬州建设取得明显成效。人民群众对社会治安满意和基本满意率连续五年保持在96%以上。贯彻落实《信访条例》，全市信访总量进一步下降。基层人民调解工作得到加强。安全生产和公共安全的基础工作不断强化。初步建立突发公共事件应急管理体系。民族、宗教工作在构建和谐社会中发挥了积极作用。国防教育、国防后备力量建设和双拥、优抚安置工作继续加强，实现“全国双拥模范城”五连冠。

六、服务型政府建设不断推进

坚持科学民主依法行政。认真执行市人大及其常委会的决议，自觉接受市人大及其常委会的法律监督和工作监督。主动接受市政协的民主监督。积极听取各民主党派、工商联和社会各界的意见和建议。五年共办理人大代表议案和建议1402件，办理政协提案1822件，解决率不断提高。认真实施《行政许可法》，先后取消和退出行政审批事项936项。全面推进政务公开，积极开展“阳光工程—规范事权行动”。实行行政审批“两集中、两到位”，启用网上审批、在线服务，提高了行政服务效能。政府公开电话和“寄语市长”的回复率均在90%以上。加强公共财政建设，新增财力重点用于公共产品、公共服务。每年都确定并实施一批为民办实事项目。认真落实党风廉政建设责任制。大力加强政府自身建设，坚持勤政为民、廉洁从政。抓好重点领域、重点行业的纠风和反腐败工作，查处了一批违纪违法案件。

各位代表，经过五年奋斗，我市经济社会发展跃上了新的平台。今天的扬州，经济充满活力，文化富有特色，城市秀美宜居，社会安定和谐，人民倍感自豪。这些成绩的取得，是全市人民在中共扬州市委领导下团结奋斗的结果，是市人大、市政协及各位人大代表、政协委员有效监督的结果，是历届政府打下良好基础和老同志关心支持的结果，也是各民主党派、工商联、无党派人士、人民团体、驻扬部队和社会各界共同努力的结果。在此，我代表扬州市人民政府向全市人民、向关心支持扬州发展的同志们、朋友们，表示崇高的敬意和衷心的感谢！

在肯定成绩的同时，我们也清醒地看到，工作中还存在一些不容忽视的问题。主要表现在：转变发展方式、调优产业结构、化解资源环境约束的压力还很大；工业集聚度不高，重大项目不多，自主创新能力还不强；城市布局、基础设施和功能建设与经济社会发展还不相适应，城市品质有待进一步提升；城乡居民收入水平和生活质量还不高，促进创业就业、统筹城乡发展等方面的任务还很重；转变政府职能、提高办事效率和改进工作作风还有许多工作要做。这些问题我们必须高度重视，认真研究并切实加以解决。

今后五年政府工作的总体要求

今后五年，是“全面达小康、建设新扬州”的关键阶段，也是我市信息化带动、工业化转型、城市化加速、市场化完善和国际化提升的重要时期。我们必须充分认识全面建设更高水平小康社会所肩负的使命和责任，充分把握新一轮发展所面临的机遇和挑战，充分凝聚全市人民的智慧和力量，以更加解放的思想、更加开放的胸怀、更加创新的举措和更加扎实的作风，全力推动扬州在科学发展的道路上迈出更加坚实的步伐。

今后五年发展的指导思想是：认真贯彻党的十七大精神，高举中国特色社会主义伟大旗帜，以邓小平理论和“三个代表”重要思想为指导，以科学发展观为统领，着力转变发展方式，提高城市竞争力；着力加快名城建设，提高城市美誉度；着力改善民生，提高人民群众幸福感；着力统筹协调发展，提高社会和谐程度；着力转变政府职能，提高公共服务水平，又好又快地实现“全面达小康、建设新扬州”的奋斗目标。

今后五年的主要指标安排：全市地区生产总值年均增长13%左右。财政总收入、一般预算收入年均分别增长16%和18%。固定资产投资年均增长20%。社会消费品零售总额年均增长15%以上。城市居民人均可支配收入、农民人均纯收入年均分别增长10%和9%。万元地区生产总值综合能耗年均递减4%。化学需氧量、二氧化硫排放量年均分别递减3.7%和3.1%。

今后五年要实现以下奋斗目标：

——经济实力再上新台阶。到2012年，地区生产总值在2007年的基础上实现翻番，达到2600亿元；工业建成石油化工、汽车船舶、装备制造、太阳能光伏4个千亿级产业，全市工业总产值突破1万亿元。累计注册外资实际到账100亿美元，新增民资注册资本金3000亿元。新农村建设取得重大进展。基本形成以先进制造业为主体、高新技术为先导、现代高效农业为基础、现代服务业为支撑的产业发展新格局。

——社会建设取得新进展。办好人民满意的基础教育，基本实现教育现代化，高中阶段毛入学率稳定在95%以上，形成较为完善的现代国民教育、终身教育和优质教育体系。城乡居民公共卫生医疗服务体系覆盖率达100%。公共文化服务体系比较完善，文化扬州建设取得明显成效。各项社会事业协调发展。全民素质显著提高。

——城市品质得到新提升。重大基础设施建设取得新突破。建成扬州泰州机场、宁启铁路复线和新宁通、江海高速公路扬州段，以及新淮江公路、安大公路；开工建设五峰山过江通道和淮扬镇铁路，初步确立区域交通枢纽地位。基本形成结构合理、功能互补、各具特色的城镇体系，城市化率达58%。瘦西湖及扬州历史城区申遗工作取得阶段性成果，创成“国家生态市”和“中国最佳旅游城市”，进一步彰显人文、生态、宜居、精致的城市特色。

——人民生活水平实现新提高。到2012年，城市居民人均可支配收入达24000元，农民人均纯收入突破1万元。全民创业深入推进，社会就业更加充分。覆盖城乡居民的社会保障体系比较完善，人人享有基本生活保障和基本医疗卫生服务。社会安定有序、和谐稳定。人民群众的精神文化生活更加丰富，民主权利得到进一步保障，幸福感、满意度普遍增强。

实现上述目标，扬州的经济将更加繁荣、社会将更加和谐、城市将更加精致、人民将更加幸福，“古代文化与现代文明交相辉映的名城”、“更加富裕、文明、秀美的新扬州”将成为现实。

2008年政府工作任务

今年是贯彻落实党的十七大精神、全面建设更高水平小康社会的重要一年，是改革开放30周年和北京奥运会举办之年，也是新一届政府任期的开局之年。做好今年工作责任重大，意义深远。面对科学发展的更高要求和宏观调控的新形势，我们必须坚定信心，锐意进取，统筹兼顾，求真务实，努力促进经济社会又好又快发展。

全市经济和社会发展的预期性指标为：地区生产总值增长14%，其中一产增长5%，二产增长16%，三产增长15%。财政总收入增长18%，其中一般预算收入增长20%。全社会固定资产投资增长25%。社会消费品零售总额增长16%。城市居民人均可支配收入增长12%，农民人均纯收入增长10%。居民消费价格涨幅不高于全省平均水平。城镇登记失业率控制在4%以内。约束性指标为：万元地区生产总值综合能耗下降4%。化学需氧量、二氧化硫排放量分别削减3.7%和3.1%。

为实现上述目标，主要抓好八项工作：

一、转变经济发展方式，提高发展的质量和效益

加快发展先进制造业。壮大提升石化、汽车及零部件、船舶、装备制造等支柱产业，产值增长30%以上，进一步形成产业集聚优势。大力扶持以太阳能光伏、半导体照明为代表的“三新”产业发展。以高新技术和先进适用技术改造、提升现有产业，提高产品附加值。全年规模以上工业产值、增加值、利税分别增长30%、20%、30%。全力推进“百亿元企业、百亿元特色产业集群”工程，年内产值过10亿元的企业达40个、过50亿元的企业达5个，18个特色产业群总产值突破1200亿元。努力增加工业有效投入，全年完成工业投资700亿元。实施亿元以上项目300个，其中10亿元以上项目20个。大力实施质量兴市战略。全年新增省级品牌产品40个以上，中国名牌产品和国家免检产品6个，中国驰名商标1件，获国家专业技术标准授权机构6家。进一步做大做强建筑业，全年建筑业施工产值增长20%以上。

大力发展现代服务业。加快数字扬州建设，基本实现城区宽带无线上网全覆盖，实施一批电子政务、电子商务和数字社区项目。信息服务产业基地基础设施投入10亿元以上，引进入驻企业20家。全市信息服务业销售收入增长30%以上。加快构建物流运输、公共信息、存储配送等服务平台，全面推进港口、石化、公铁水和商贸物流园区建设。进一步优化金融生态环境，引导银行增加有效信贷投入和新的金融产品，积极争取新的金融、保险机构落户扬州。加大旅游整体宣传推介力度，加强境内外旅游促销，积极开拓日韩、东南亚等客源市场。完善古城、古运河、瘦西湖新区游览线。整合市、县旅游资源，拓展一批特色旅游项目。改善旅游配套设施，提升服务水平。积极申报“中国最佳旅游城市”，瘦西湖风景区创建成5A级景区。高邮争创“中国优秀旅游城市”。全年接待游客总人数和旅游收入均增长20%。大力推进“万村千乡”市场工程，繁荣农村消费市场。引导专业市场发展，培育壮大特色市场群。放大“老字号”和“三把刀”品牌效应，提高传统服务业的运营质态。加快城市新区商贸服务业发展。鼓励发展特色会展、创意产业和各类中介服务业。稳步健康发展房地产业。全年服务业增加值占地区生产总值的比重提高1个百分点。

全力推动自主创新能力建设。继续实施“双创”、“三重”政策，加大“三新”产业扶持力度。组织实施省级以上科技计划项目400项，全年培育省级以上高新技术企业58家，新增国家级检测中心1家、省级企业技术开发中心和工程研究中心5家。高新技术产业产值增长40%。深化与中科院、南京大学、扬州大学等科研院所和高校的产学研合作，加快国家级产业基地的建设，发挥各类创新平台的作用。依托开发园区，建设、完善各类科技创业服务中心和孵化器。加快创新人才引进和培养，制订激励高层次人才创业创新的政策措施。加强企业家队伍建设，提高企业家经营管理能力。

坚决抓好节能减排工作。优先发展高技术、高效益、低污染、低能耗产业，严格执行项目开工建设“六项必要条件”，切实限制高能耗、高物耗项目。建立健全节能减排统计公布体系和评价考核体系，严格实行责任追究制、一票否决制和履职报告制度。抓好重点耗能行业和企业的节能减排工作。对115家重点耗能大户加强监控，淘汰落后产能设备1200台套以上。全部拆除市区禁燃区4吨以下、各县（市、区）2吨以下燃煤设施。完成省下达的小化工企业关停任务。加快城区工业企业“退城进园”步伐。启动循环经济重点项目工程。强制清洁生产实施率达100%。

二、坚持城乡统筹，扎实推进新农村建设

促进城乡一体化发展。按照城乡发展规划、基础设施、公共服务、就业社保和社会管理一体化的要求，加大公共财政对农村的投入，推动以工促农、以城带乡长效机制的建设。重点统筹城乡规划建设，完善县城镇和重点中心镇规划，完成1100个村庄建设规划的编制。重点加强县乡公路与国省干线的网络化对接，加强区域集中供水管网等公用设施建设，促进基础设施向农村延伸，生产要素向农村流动，公共服务向农村拓展，现代文明向农村传播。

全面提升县域经济综合实力。深入实施“强县强镇、三年倍增”计划。统筹沿江沿河开发，加快建设市域环路经济带，优化生产力布局，发展板块经济和特色产业。增强各类园区的集聚功能，调优产业结构，提升工业化水平。完善城镇体系，加快城镇化进程，增强县城镇、重点中心镇的辐射带动能力。全年县域地区生产总值、财政收入增幅高于全市平均水平。

大力发展现代高效农业。以高效蔬菜、花木茶果、特色畜禽、特色水产等为重点，推动高效农业规模化。着力培育一批产值超10亿元的农业特色产业，全年新增高效农业面积40万亩以上。加强农产品质量安全监管，新增一批无公害、绿色、有机农产品。提高“菜篮子”工程的建设水平。加快发展现代农业服务业，提高生产资料、信息、机械、技术和农产品流通等服务水平。充分发挥海峡两岸(扬州)农业合作试验区和现代农业园区的作用，大力吸引外来资本进入农业领域。全年农业利用外资1.5亿美元，民资和工商资本40亿元。大力发展农村“三大合作”组织，不断提升农业产业化、市场化水平。全年新增市级以上龙头企业10家，销售过10亿元企业3家。

着力改善农村生产生活环境。深入推进新农村建设“十大工程”和新一轮“五件实事”。建设改造农村公路500公里、大中桥梁30座，完成农村路桥投资3.2亿元。全面落实新型农村公路养护体制。抓好水利血防、小水库除险加固、圩区治理等工程建设。调整优化村庄布局，继续抓好10个农民集中居住区的试点。以“三清一绿”为重点，深入开展农村环境综合整治。扩大区域集中供水范围，实施农村“二次改水”工程，保障农村居民饮水安全。推进乡镇垃圾集中处理。新增农村无害化户厕5万个。新建户用沼气池1万个、中小型沼气工程10座以上。认真开展新农村示范村和全面小康村创建活动。切实做好新一轮经济薄弱村和低收入农户帮扶工作。

三、推进改革开放，增强发展的动力和活力

加大重点领域和关键环节的改革力度。加快国有集体企业改革扫尾和企业内部制度改革，支持有条件的企业通过重组和改造提升竞争力。落实鼓励企业上市的政策措施，推动企业股份制改造，培育更多的上市后备企业。力争年内顺大、诚德等4家以上企业上市，宝胜股份等上市企业实现再融资，引导更多的企业利用资本市场加快发展。培育发展担保机构，积极引进风险投资、创业投资基金来扬开展业务。充分发挥现有融资平台的作用，构建基础设施、公共设施多元化投入、市场化运作的新机制。健全土地、技术等要素市场，规范发展产权交易市场。深化农村综合改革，继续搞好乡镇机构改革试点。加快农村信用合作社改革。做好政策性农业保险工作。稳步推进文化、卫生、教育和社会公用企事业等方面改革。组织实施第二次全国经济普查工作。

强化园区开发建设。按照功能创新、科技创新、管理创新的要求，调整完善新一轮园区总体发展规划，做大做强园区主导产业，推动产业集聚集约发展，提高单位面积投资强度和产出效益。继续加强园区基础设施建设，不断完善园区政务、商务、生活服务配套功能，增强园区的综合优势。转变园区发展方式，鼓励创建循环经济园区。加强出口加工区建设，放大综合效应。

全面提升对外开放水平。深化产业招商，引导外资投向石油化工、装备制造、太阳能光伏、半导体照明等重点产业，加快形成优势产业链。强化企业招商，鼓励重点骨干企业和成长型企业瞄准跨国公司、行业龙头，扩大合资合作，提升发展水平。鼓励现有的外资企业增资扩股、参与改组改造。加强招商重点区域境外办事处建设，深入开展驻点招商、代理招商。拓展节庆招商，办好2008“烟花三月”国际经贸旅游节，积极开展境内外招商推介活动，扩大信息源、项目源。全年协议利用外资45亿美元，注册外资实际到账15亿美元。优化口岸快捷便利服务，加强出口产品品牌建设，提高产品质量和附加值。鼓励企业“走出去”参与国际竞争与合作，扩大对外工程承包和劳务合作。外贸出口、外经营业额分别增长15%和20%。

推动民营经济健康快速发展。落实支持民营经济发展的政策措施，加强对民营企业的社会化服务，进一步营造民营经济加快发展的良好环境。引导民营企业加快机制创新，建立现代企业制度。鼓励民营企业加快技术创新，提高产品档次和技术含量。支持有条件的民营企业实施集团化发展战略，推动企业做大做强。强化民资招商，大力引进高新技术项目和特色优势项目。新开工建设3000万元以上项目300个。全年新发展民营企业1万户以上，新增民资注册资本金400亿元。

四、完善城市功能，提升城市品质

进一步优化城市空间布局。主动对接国家和省主体功能区规划，合理设置市域功能区和产业布局。接轨长三角一体化和宁镇扬经济板块，完善区域综合交通等重大基础设施建设规划。实施“一体两翼”发展规划，形成“一体两翼”城市发展新格局。开展城市总体设计研究，做好城市综合交通规划、广陵新城等分区、片区的控制性详规，以及重点地段的城市设计。切实加强规划后的管理。

加快重大基础设施建设。全面开工建设宁通高速六合至江都段、江海高速江都段，加快安大公路三垛以北段、新淮江公路、金宝南线等国省干线公路建设。继续推进京杭运河“三改二”工程。开工建设施桥、邵伯船闸扩容等工程。加快扬州港5号码头建设。配合建设宁启铁路复线及电气化改造。推进扬州泰州机场等重大项目立项工作。加强城市防洪、城乡电网、给排水、供气、通信等基础设施的统筹建设。启动第五水厂一期工程。

加大主城区建设力度。加快主城区与江都、仪征的连接，年内实施文昌东路延伸和仪扬路、扬冶路、扬江路改造等项目，建设新廖家沟大桥。完善主城区路网体系，新建、改造北外环路、开发路、竹西路、长征路、解放北路等道路，做好扬子江南路、文昌中路、淮海路、渡江南路等路段的街景美化工作。建设运河北路古运河大桥、太平河大桥。综合治理七里河、新城河等河道。开工建设乌塔沟分洪道工程。加快新城西区、广陵新城、蒋王片区、蜀冈片区等开发建设，进一步增强城市的商务、休闲等功能。

推进古城保护。编制瘦西湖及扬州历史城区保护规划。积极做好瘦西湖及扬州历史城区申遗工作和中国大运河联合申遗的牵头工作。实施“双东”历史街区个园东扩、壶园复建等“一线十点”项目建设，加快教场商贸民居民俗文化核心区建设，有序推进南河下片区整治。完善南门遗址公园，建设北门遗址公园。丰富古运河文化公园内涵，打造护城河、小秦淮河等亲水景观带。推进瘦西湖新区建设，规划建设万花园二期、宋夹城文化景区等项目。办好第二届世界运河名城博览会，进一步彰显运河名城特色。

提高城市管理水平。实施数字化城管二期工程，进一步扩大覆盖范围，提高处置能力。整治违法建设、占道经营、乱贴乱画、乱倒垃圾等突出问题，进一步加强背街小巷的管理。全面实行市区主要道路市场化保洁，试行开放式公园、公共绿地等市政公用设施第三方托管。加强环卫设施建设，提升城市管理配套能力。引导公众参与城市管理，全面改善市容市貌。

加强生态环境建设和资源节约利用。以创建“国家生态市”和“国家生态园林城市”为抓手，加快长江、京杭运河等重要绿化风光带和农田林网建设，新增造林面积10万亩，新增市区绿化面积150万平方米。加强水资源的节约利用和保护，继续开展集中式饮用水源地专项整治，实施南水北调生态功能保护区一期工程。开工建设六圩污水处理厂二期工程，完善城区及工业园区污水管网。建设垃圾焚烧发电厂。宝应争创国家生态县，江都、仪征争创国家环保模范城。全市30%的乡镇、村创成环境优美乡镇、生态村。强化土地管理和调控，严格实行耕地保护责任制，推进集约节约用地，严禁违法违规用地。

五、发展社会事业，促进经济社会协调发展

优先发展教育事业。积极推进素质教育和教育现代化建设工程，办好人民满意的教育。着力改善农村中小学办学条件，扩大利用优质教育资源，推动义务教育高位均衡发展。高中阶段毛入学率稳定在95%以上。大力发展职业教育，进一步做大规模、做强特色、做优品牌。开工建设扬州高等职业学校（筹）一期工程，完成扬州高级职业中学搬迁改造。提升教育信息化水平，推进数字化校园建设。加强教师队伍建设，提高师资水平，改善教师待遇。实行义务教育阶段免收学杂费和免费提供教科书。全面化解农村义务教育阶段学校债务。

推动文化繁荣与发展。积极发展公益性文化。开工建设市文化艺术中心，新改建20个乡镇文化站和200个村文化室。推进文化博览城建设。开工建设马可·波罗纪念馆、扬州学派史料陈列馆等，基本建成佛教文化博览馆。认真做好第三次全国文物普查工作。公布第四批市级文物保护单位和首批市级非物质文化遗产名录。大力发展文化产业，形成区域性特色文化产业群，市工艺美术集团争创国家级文化产业示范基地。办好第六届市少儿艺术节和“市民日”等节庆活动。完善文化事业、文化产业、文化人才的鼓励扶持政策，重点支持地方剧种出精品、出人才。

加强公共卫生服务体系建设。全年新建城乡社区卫生服务中心20个、社区卫生服务站500个，居民社区卫生服务中心（站）覆盖率城区达90%以上、农村80%以上。实行二级以上医院与社区卫生服务中心的对口支援、双向转诊，加强城乡社区医技人员培训，提高医疗卫生服务水平。做好城乡爱国卫生工作，建成省级卫生镇2个。加强公共卫生监督管理。突出抓好重大疾病的防治，不断提高传染病预防控制能力。完成苏北医院、市妇幼保健院改造。

促进其他社会事业发展。稳定低生育水平，提高出生人口素质，计划生育率保持在97%以上。加强公共体育设施建设与管理，深入开展全民健身运动。办好奥运圣火在扬传递活动、鉴真国际马拉松赛和市第十一届运动会。新建市游泳馆。基本完成主城区有线数字电视整体转换工作。加快建设以“组组通”为基础的农村广播电视服务体系。切实加强网络文化建设与管理。重视开展社会科学研究。认真做好二轮修志、档案等工作。

六、着力改善民生，让发展成果惠及于民

促进创业和就业。完善自主创业、自谋职业和灵活就业的鼓励政策，强化创业辅导培训、融资等服务，优化创业环境，支持更多的劳动者成为创业者。加快公共就业服务体系建设，健全城乡一体化的劳动力市场。推进“充分就业社区”创建工作。积极帮扶大中专毕业生等新成长劳动力、“零就业”家庭和就业困难人员就业。全年采集就业岗位9万个，新增就业4.3万人。健全失地农民就业和失业专项登记制度，帮助城镇规划区内失地无业农民就业。加大企业下岗人员、失地农民的技能培训，提高就业成功率。全年农村劳动力开发就业培训4万人。贯彻落实《劳动合同法》，维护劳动者合法权益。

继续增加城乡居民收入。坚持富民优先，建立和完善企业职工、机关事业单位工作人员和离退休人员等工资正常增长的长效机制。逐步提高居民收入在国民收入分配中的比重，提高劳动报酬在初次分配中的比重。着力提高低收入者收入，逐步提高扶贫标准和最低工资标准。继续调整企业退休人员基本养老金。创造条件让更多群众拥有财产性收入。通过发展高效农业、农村劳动力转移、土地流转收益等途径，不断提高农民收入。

健全社会保障体系。完善城镇职工养老保险制度，净增缴费人数3万人。积极开展新型农村社会养老保险试点。不断扩大医疗保障覆盖范围，新增城镇职工参保3万人，城镇居民参保4万人。完善医保政策，降低医疗费个人自付比例。新型农村合作医疗覆盖率稳定在95%以上，参合最低标准由每人50元提高到100元。推进农民工“平安计划”。建立健全被征地农民基本生活保障制度，严格执行即征即保。完善城乡低保标准自然增长机制，切实做到应保尽保。农村五保户集中供养率达80%。市区设立困难群体临时救助资金，加强对低保边缘群体的应急救助。

切实解决影响民生的突出问题。着力解决城镇居民住房困难，扩大限价商品房和廉租房保障范围。对符合条件的特困家庭，给予廉租房租金补贴或安排廉租房；对符合条件的低收入家庭，提供限价商品房或给予购房补贴。市区新开工建设限价商品房25万平方米，解困解危房5万平方米。积极开展教育帮扶工作。确保经济困难家庭、进城务工人员子女和农村留守儿童平等接受教育，对高中生、中等职业技校特困生发放助学金，确保所有考上大学的学生不因贫辍学。扩大“宏志班”办学规模，市区新招中小学宏志班12个。加强惠民医院、惠民门诊建设，完善对困难群体的医疗救助制度。对低保特困家庭中的重症患者减免医疗费用。大力发展扶老、助残、救孤等福利事业。坚持为民办实事制度，着力解决人民群众关心的老小区整治、农贸市场建设和公共交通等热点难点问题。

七、加强社会管理，推进和谐社会建设

深入开展平安扬州建设。健全社会治安防控体系，落实社会治安综合治理各项措施，积极防范和依法打击违法犯罪活动。建立和完善人民群众诉求表达机制，进一步落实信访工作责任制，提高矛盾调处工作的针对性和有效性，着力化解人民内部矛盾。完善司法救助机制，加大法律援助力度，促进社会公平正义。建立安全生产、道路交通、食品药品、危险品和消防安全等长效监管机制。完善市应急指挥中心建设，加强对突发公共事件的预防和处置，切实保障人民生命财产安全。

加强社会组织建设。大力支持社会团体、行业组织、民办非企业单位和城乡基层自治组织的发展，发挥其协助政府进行社会管理和提供公共服务的作用。加强行业协会建设，促进行业自律、规范发展。理顺社区管理体制和运行机制，健全社区民主自治制度。加强社区公共服务设施建设，完善社区管理服务功能，提高居委会、村委会规范化建设水平，把城乡社区建设成为管理有序、服务完善、文明祥和的家园。

加强精神文明建设。深入开展社会主义荣辱观教育，进一步加强社会公德、职业道德、家庭美德、个人品德建设，全面推进群众性精神文明创建活动，不断促进社会主义核心价值体系的形成。加强社会信用体系建设，整体推进政务诚信、商务诚信和社会诚信。大力弘扬“开放、包容、创新、精致”的扬州精神，营造共建共享和谐的社会环境。做好国防动员工作，支持驻扬部队建设。深入开展国防教育和双拥活动，争创一批省级“双拥模范城（县、区）”。

八、加强政府自身建设，提高公共服务能力和水平

完善公共服务体制。加强公共财政建设，优化财政支出结构，确保新增财力重点用于教育、文化、卫生、科技和社保等公共产品和公共服务。健全国库集中支付、政府采购和部门综合预算制度，继续推进非税收入管理体制改革。扩大政府重点投资项目代建制实施范围，提高财政资金使用效益。继续推进政企分开、政资分开、政事分开、政府和市场中介组织分开，切实履行社会管理和公共服务职能。深化行政审批制度改革，进一步简化审批程序、减少审批环节，强化行政办事服务中心建设，完善相对集中的行政审批机制。加强电子政务建设，扩大政府网上服务事项，为市民提供公开、透明、高效的网上服务。

推进民主法制建设。认真执行市人大及其常委会的各项决议、决定，依法接受市人大及其常委会的法律监督、工作监督。支持市政协履行职能，自觉接受市政协的民主监督。定期向市人大及其常委会报告工作和向市政协通报情况。认真做好人大代表议案、建议和政协提案的办理工作。健全与各民主党派、工商联、无党派人士的联系制度，充分发挥工会、共青团、妇联等群众团体的作用。积极推进各种形式的基层民主，进一步提高人民群众有序政治参与的程度和水平。建立民主科学的公共决策机制，实施重大决策事项公示、听证和专家咨询制度，提高行政决策的科学性。继续深入推进政务公开，建立健全新闻发言人制度，保障人民群众的知情权、参与权、表达权和监督权。广泛开展法制宣传教育，认真组织实施“五五”普法。坚持依法行政，规范行政权力。严格执行行政问责制度，做到有责必问、有错必纠。

强化政府作风建设。教育引导全体公务员特别是各级领导干部进一步解放思想，树立强烈的开放意识、创新意识、诚信意识和服务意识。加强学习型机关建设，不断提高公共服务的能力和水平。认真践行执政为民宗旨，着力解决改革发展稳定中的重大问题和人民群众生产生活中的实际问题，切实提高政府机关的执行力。加强发展软环境建设，深入开展“阳光工程—规范事权行动”，定期组织基层和群众评议政府机关工作。牢记“两个务必”，倡导勤俭节约，反对铺张浪费，建设节约型政府。严格执行党风廉政建设责任制，更加注重治本、预防和制度建设。强化重点领域重点问题的专项整治，坚决纠正损害群众利益的不正之风。加强行政监察、审计监督，加大标本兼治、综合治理和查处违法违纪案件的力度，努力建设勤政、高效、务实、廉洁的政府。

各位代表，本次人代会将开启扬州又一个五年的崭新征程。新的蓝图无限美好，新的目标催人奋进。让我们在中共扬州市委的领导下，紧紧依靠全市人民，同心同德，再接再厉，求真务实，开拓创新，为全面建设更高水平的小康社会而努力奋斗！
